# Downregulation of Notch4 – a prognostic marker in distinguishing oral verrucous carcinoma from oral squamous cell carcinoma^☆^

**DOI:** 10.1016/j.bjorl.2017.09.005

**Published:** 2017-10-31

**Authors:** M.K. Harishankar, A. Mathan Mohan, A. Vinod Krishnan, Arikketh Devi

**Affiliations:** a SRM University, School of Bioengineering, Department of Genetic Engineering, Kattankulathur, India; b Karpaga Vinayaga Institute of Medical and Dental Sciences, Department of Oral and Maxillofacial Surgery, Oral Cancer Foundation, Kancheepuram, India

**Keywords:** Oral verrucous carcinoma, Oral squamous cell carcinoma, Notch4, Prognostic marker, Carcinoma verrucoso oral, Carcinoma de células escamosas oral, Notch4, Marcador prognóstico

## Abstract

**Introduction:**

Oral verrucous carcinoma is a special form of well-differentiated squamous cell carcinoma which possesses specific clinical, morphologic and cytokinetic features that differ from other types of oral cancers and hence diagnosis requires immense experience in histopathology. Hence it is certainly important to distinguish such a lesion from other oral tumors as treatment strategies vary widely between them.

**Objective:**

In search of a critical diagnostic marker in distinguishing oral verrucous carcinoma from oral squamous cell carcinoma, Notch4 receptor, one of the key regulatory molecules of the Notch signaling family has been aberrantly activated in the progression of several types of tumors. However its function in oral verrucous carcinoma remains unexplored. Thus the present study aims in determining the differential expression pattern of Notch4 in oral verrucous carcinoma and oral squamous cell carcinoma.

**Methods:**

Ten patients reported positive for oral cancer (5 patients with oral verrucous carcinoma and 5 patients with oral squamous cell carcinoma). Five normal tissue samples were also obtained and evaluated for clinicopathological parameters and immunohistochemistry, western blotting and real time polymerase chain reaction for Notch4 expression.

**Results:**

Our results reveal that the expression of Notch4 was considerably high in oral squamous cell carcinoma lesions compared to normal tissue, whereas in oral verrucous carcinoma, irrespective of the clinicopathological features, complete regulação descendente of Notch4 was observed.

**Conclusions:**

These preliminary findings strongly support the fact that Notch4 is downregulated in oral verrucous carcinoma and could be considered as a suitable prognostic marker in distinguishing oral verrucous carcinoma from oral squamous cell carcinoma. This distinguishing marker can help in improving therapeutic options in patients diagnosed with oral verrucous carcinoma.

## Resumo

**Introdução:**

O carcinoma verrucoso de cavidade oral é uma forma especial de carcinoma de células escamosas bem diferenciada que tem características clínicas, morfológicas e citocinéticas específicas que diferem de outros tipos de cânceres orais. Por essa razão, o diagnóstico requer grande experiência em histopatologia. Portanto, é certamente importante distingui-lo de outros tumores orais, pois as respectivas estratégias de tratamento variam muito.

**Objetivo:**

Em busca de um marcador de diagnóstico crítico na distinção entre o carcinoma verrucoso e o carcinoma de células escamosas de cavidade oral, o receptor Notch4, uma das principais moléculas reguladoras da família de sinalizadores Notch, foi ativado de maneira anormal na progressão de vários tipos de tumores. No entanto, sua função no carcinoma verrucoso permanece inexplorada. Assim, o presente estudo tem como objetivo determinar o padrão de expressão diferencial de Notch4 no carcinoma verrucoso e de células escamosas de cavidade oral.

**Método:**

Dez pacientes tiveram resultado positivo para câncer oral (cinco pacientes com carcinoma verrucoso e cinco pacientes com carcinoma de células escamosas) e cinco amostras normais foram também obtidas. Além da avaliação dos parâmetros clínico-patológicos, foram feitos análise imuno-histoquímica, Western Blot e reação de polimerase em cadeia em tempo real para a expressão de Notch4.

**Resultados:**

Nossos resultados revelam que a expressão de Notch4 foi consideravelmente alta em carcinomas de células escamosas em comparação com os tecidos normais, enquanto que no carcinoma verrucoso, independentemente das características clínico-patológicas, observou-se *regulação descendente* completa de Notch4.

**Conclusão:**

Esses achados preliminares apoiam fortemente o fato de que Notch4 estava regulado para baixo no carcinoma verrucoso oral e poderia ser considerado um marcador prognóstico adequado para distinguir entre carcinoma verrucoso e carcinoma de células escamosas de cavidade oral. Esse marcador distintivo pode ajudar a melhorar as opções terapêuticas em pacientes com diagnóstico de carcinoma verrucoso oral.

## Introduction

Oral Verrucous Carcinoma (OVC), typically representing a rare variant of well differentiated squamous cell carcinoma is considered to be a non-invasive form of tumor with specific clinical, morphologic and cytokinetic features.^1^, ^2^ In general, lymph node and distant metastasis are rare in OVC but the large size and involvement of bone structures, renders it locally aggressive if not treated properly.^3^ Though OVC has unique pathological characteristics, it's very challenging for the pathologist to differentiate among oral cancer subtypes because accurate diagnosis requires adequate tumor samples as well as experienced clinicians.^4^ Treatment of OVC still remains controversial because of their extensive nature mimicking an invasive cancer and hence identification of a critical diagnostic marker which could discriminate the components of Oral Squamous Cell Carcinoma (OSCC) from OVC is crucial to evaluate the clinical significance of OVC.^5^ Therefore, the need of the hour is to identify a definite marker for OVC which could be used effectively to diagnose and treat OVC.

Notch signaling pathway is one of the cell to cell communications signaling pathways that promotes a vast array of regulatory functions such as cell proliferation, differentiation and apoptosis.^6^ The elevated expression of the notch signaling molecules (Notch 1–4 receptors, Delta like 1, Delta like 3, Delta like 4, Jagged 1 and Jagged 2 ligands) has been considered to be one of the critical event in several malignancies.^7^, ^8^, ^9^ Importantly, accumulative evidence has shown that constitutive activation of Notch4 receptor, one of the key receptor molecules of the Notch signaling family has been associated with several cancer pathogenesis. However, its function as oncogene or tumor suppressor gene is cell context specific.^10^, ^11^

In our previous study, we found that Notch4 plays an important role in the pathobiology of OSCC and hence this study has been targeted to analyze the expression of Notch4 among OVC and OSCC.^12^ To achieve this, the expression of Notch4 was analyzed on the tumor sections of OVC and OSCC with varied clinicopathological parameters. The purpose of the study was to determine the differential expression pattern of Notch4 between the major subtypes of oral cancer such that a reliable diagnostic marker could be established for the improved treatment of OVC patients.

## Materials and methods

### Patients sample with clinicopathological parameters

A total of 15 post-surgical oral cancer samples which includes 5 samples reported positive for OSCC and 5 samples with OVC were collected along with 5 normal oral mucosa samples from individuals who underwent surgery for benign oral and maxillofacial conditions from the Department of Oral and Maxillofacial Surgery, Karpaga Vinayaga Institute of Dental Sciences, India. All the samples were divided into two parts: one part was fixed in 10% buffered formaldehyde solution and the other part was frozen immediately and stored in −80 °C until use. Information on the various clinical parameters (gender, age, site of tumor and TNM staging) were obtained from medical records. The study was approved by the Institutional Ethical Committee (490/IEC/2013).

### Immunohistochemical analysis

Immunohistochemical analysis for the tumor sections were performed as previously described.^12^ Primary antibodies used were as follows: Notch4 (sc-8646), GAPDH (sc-47724). After incubating the samples with HRP conjugated secondary antibodies, the slides were examined under a light microscope and the results were categorized as high, moderate and mild and negative expression based on high versus low antigen expression. All the antibodies were purchased from Santa Cruz Biotechnology, USA.

### Immunoblotting

Western blot analysis was performed on total proteins harvested from the tissue sections using lysis buffer. Briefly, the separated proteins were transferred to nitrocellulose membrane (Amersham Protran, GE Healthcare Life Sciences, Germany) and blocked with 3% BSA in Tris Buffered Saline with 0.1% Tween 20 (TBST) followed by overnight incubation with anti-Notch4 (sc-8646) or anti-GAPDH (sc-47724) at 4 °C. The membrane was incubated with suitable horse radish peroxidase-labeled secondary antibodies at 37 °C for 1 h. The blots were then developed using DAB (Sigma) as per manufacturer's protocol. Densitometric analysis was performed on the blots.

### RT-PCR analysis

Total RNA was isolated from frozen tissue samples using Trizol (Merck) and quantified using the Nanodrop system (Nanodrop lite spectrophotometer, Thermo Scientific). cDNA was prepared from 1 μg of total RNA using M-MuLV reverse transcriptase (New England Biolabs Inc.). Prepared cDNA was subsequently subjected to PCR amplification using the following primers: Notch4: forward: 5′-CCACTAGGCGAGGACAGCATT-3′; reverse: 5′-CAACTCCATCCTCATCAACTTCTG-3′; β actin: forward: 5′-AGAGCTACGAGCTGCCTGAC-3′; reverse: 5′-GGATGCCACAGGACTCCA-3′. The amplified PCR products were visualized using agarose gel electrophoresis by ethidium bromide staining. All the samples were normalized with β actin using densitometric analysis.

### Statistical analysis

Statistical analysis of the expression of Notch4 between OVC and OSCC patients were analyzed by Student's *t*-test using the Graph pad online software (www.graphpad.com/quickcalcs/ttest1). All the experiments were repeated thrice and the statistical tests were performed at a significant level of *p* < 0.05.

## Results

**Figure 1:**
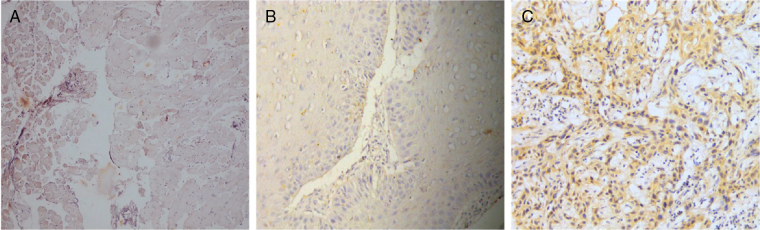
Immunohistochemical expression of Notch4 protein in specimens of normal mucosa and subtypes of oral cancer (OVC and OSCC) (20× magnification). (A) Immunoreactivity of Notch4 in normal mucosa of the oral cavity. (B) Immunoreactivity of Notch4 in OVC. (C) Immunoreactivity of Notch4 in OSCC.

**Figure 2:**
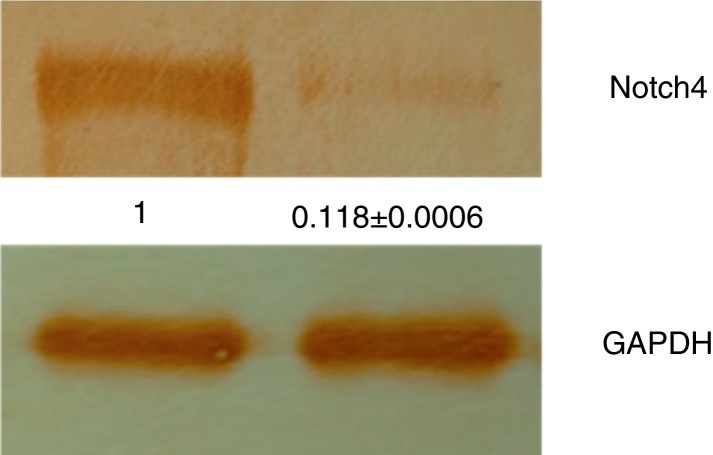
Total proteins isolated from OVC and OSCC tissue samples were analyzed by western blotting using anti-Notch4 antibody. The blots were developed using DAB, showing a high expression of Notch4 in OSCC samples whereas very poor expression was observed in OVC samples. All the experiments were performed in triplicates and the data were further analyzed by densitometry with GAPDH serving as normalizer.

**Figure 3:**
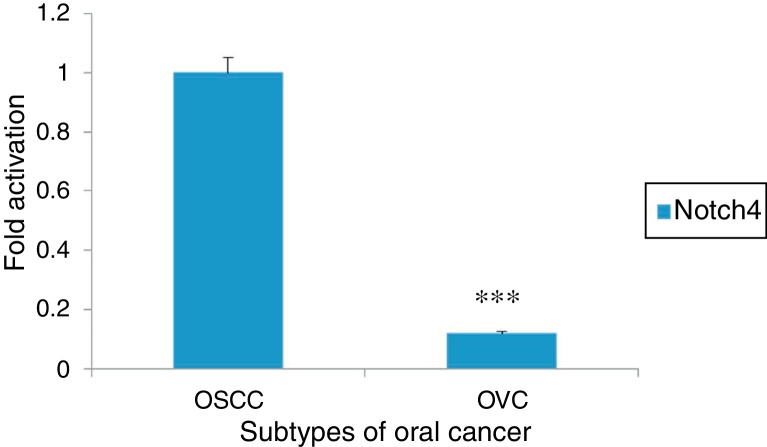
RT-PCR analysis of Notch4 in different subtypes of oral cancer (OSCC and OVC) showing a downregulation of Notch4 in OVC samples. Densitometric analysis was performed using ImageJ 1.47v software and the values were normalized to β Actin (***p < 0.001, Student's *t*-test).

The clinical characteristics of the OVC and OSCC patients have been summarized in Table 1. The mean age of the OVC and OSCC groups were 65.2 ± 3.05 years and 63.8 ± 5.19 years, respectively. Male to female ratio for both the groups was 4:1 and the primary site of the tumors was confined to buccal mucosa (3/5 in both the groups). Thus, all the major clinical parameters for both the groups selected for the study were almost identical. Although the etiological factors of OVC still remains controversial, in our study all the patients were reported with history of tobacco usage (data not shown). On performing immunolocalization for Notch4 on tumor sections it was visualized that the protein was membranous and cytoplasmic and its expression was certainly high in OSCC tumors compared to that of the normal mucosa whereas very low expression was observed in all the samples of OVC irrespective of the clinical parameters (Fig. 1). In addition, western blotting and RT-PCR data also showed that Notch4 was abundantly present in OSCC while very poor expression was seen in OVC cases (Fig. 2). Further, the densitometric analysis of the blots confirmed that Notch4 was significantly downregulated in OVC suggesting its importance in the prognosis of the tumor subtypes (Fig. 3).

**Table 1:** Clinical parameters of subtypes of oral cancer (OVC and OSCC) with the expression of Notch4 represented as high, moderate, mild and negative expression based on the intensity of the proteins visualized by immunolocalization experiments (*p* < 0.05 was considered statistically significant).

Sample no	Age (years)	Sex	Primary site of tumor	Clinical diagnosis	Notch4 expression	*p*-Value
OVC 1	61	Male	Buccal mucosa	Verrucous carcinoma	Mild	
OVC 2	70	Male	Alveolar mucosa	Verrucous carcinoma	Mild	
OVC 3	64	Male	Buccal mucosa	Verrucous carcinoma	Negative	
OVC 4	57	Male	Palate	Verrucous carcinoma	Mild	
OVC 5	72	Female	Buccal mucosa	Verrucous carcinoma	Negative	0.003
OSCC 1	68	Female	Buccal mucosa	Squamous cell carcinoma	High	
OSCC 2	70	Male	Alveolar mucosa	Squamous cell carcinoma	Moderate	
OSCC 3	52	Male	Buccal mucosa	Squamous cell carcinoma	Moderate	
OSCC 4	49	Male	Ca-Tongue	Squamous cell carcinoma	High	
OSCC 5	80	Male	Buccal mucosa	Squamous cell carcinoma	High

## Discussion

Oral verrucous carcinomas are slow-growing, exophytic, well-demarcated hyperkeratotic lesions considered to be a rare variant of squamous cell carcinomas with an occurrence rate of 2–12% among all types of oral cancer.^13^ However, OSCC is a very common neoplasm of the oral cavity representing almost 90% of the tumors of the oral cavity.^14^ In general, OSCC is considered to be a more aggressive form of tumor, often leading to metastasis which is highly uncommon in OVC. In addition, the histopathological feature of OVC remains distinct from that of other conventional carcinomas.^15^ Though distinct, an accurate histopathological diagnosis requires a skillful pathologist and a clinician with a sufficient biopsy sample with deep infiltrating portions of lesions.^16^ Hence the optimal treatment for OVC still remains controversial due to difficulties in appropriate classification of lesions and also the ability to mimic the invasive OSCC in its biologic behavior.^17^ However, a clear identification of the tumor subtypes is of great importance as treatment strategies greatly vary among the two groups.

Innumerable studies in the past have been performed in order to identify the active molecule involved in the pathogenesis of OVC. Mohtasham et al. (2013) performed histochemical analysis of p53, Ki-67, MMP-2, MMP-9 and showed that these proteins could be used in identifying the tumor invasive front that distinguishes OSCC from OVC.^18^ Similarly, several proteins such as Bcl-X Retinoblastoma (Rb) oncogene and Cyclin D1 were also found to have a differential expression in OVC.^19^, ^20^ However Ogawa et al. (2004) observed no obvious differences in p53 protein expression between VC and well-differentiated SCC in proliferative activity of tumor cells.^16^ Hence the reliability of these protein markers is questionable due to lack of uniformity in its expression pattern among individuals.^21^ Thus the differential diagnosis of OVC remains difficult and requires careful examination of the tumors.

Notch signaling pathway, one of the key cell communication pathways has an important role in maintaining the balance between cell proliferation, differentiation and apoptosis.^22^ Apart from its role in regulating biological behavior of normal cells, members of the notch family (Notch1, Notch2, Notch3 and Notch4 receptors) has also been reported to induce several types of cancer and might be considered as a potential therapeutic target in oncology.^23^, ^24^ In our previous study we demonstrated that Notch4 was upregulated in the late stages of OSCC suggesting it as a potential metastatic marker.^12^ However, its biological function as oncogene or tumor suppressor gene is purely based on cell context. For example, Clementz et al. (2011) and Nagamatsu et al. (2014) demonstrated independently the oncogenic role of Notch4 in promoting breast malignancy and suggested it as a potential therapeutic option in treating metastatic patients.^10^, ^25^ Further Ding et al. (2010) reported the upregulation of Notch4 in Salivary Adenoid Cyctic Carcinoma (SACC) and its key role in inducing SACC metastasis.^11^ Similarly Notch4 has been considered as a candidate histochemical marker in identifying hepatocellular carcinoma.^26^ Whereas in some cancer types such as renal cell carcinoma, downregulation of Notch4 has been reported.^27^ Hence it's impossible to generalize the role of Notch4 in progression of cancer. Till now we know of no reports that elucidate the tissue-specific expression of Notch4 in OVC and hence the current study was designed to identify its potential role in OVC pathogenesis. Results from the present study correlated with our previous study on the fact that expression of Notch4 was high in OSCC samples. Interestingly complete downregulation of Notch4 was observed in OVC patient samples confirming the cell and disease context specific role of Notch4 within the closely related oral cancer subtypes. Together this study provides new insight in bringing out a molecular approach in differentiating OVC and OSCC.

## Conclusions

In summary, this study was majorly focused on deriving a key diagnostic marker that could be more specific for OVC. Hence our findings confirm that depletion of Notch4 expression in OVC tissue samples could be considered as a valuable prognostic marker in differentiating OVC from OSCC, as OSCC lesions may not be distinguished clinically or may coexist with OVC. Although our results strongly supports the significance of Notch4 as a reliable prognostic marker for OVC still elaborate studies must be performed in order to derive the regulatory factors that triggers the downregulation of Notch4 such that a novel therapeutic approach for OVC could be attained.

## Conflicts of interest

The authors declare no conflicts of interest.
